# Risk factors for loco-regional recurrence in breast cancer patients: a retrospective study

**DOI:** 10.18632/oncotarget.25735

**Published:** 2018-07-13

**Authors:** Tomás Merino, Teresa Ip, Francisco Domínguez, Francisco Acevedo, Lidia Medina, Alejandra Villaroel, Mauricio Camus, Eugenio Vinés, César Sánchez

**Affiliations:** ^1^ Department of Hematology-Oncology, Pontificia Universidad Católica de Chile, Santiago, Chile; ^2^ Department of Surgery, Pontificia Universidad Católica de Chile, Santiago, Chile; ^3^ Department of Pathology, Pontificia Universidad Católica de Chile, Santiago, Chile

**Keywords:** breast cancer, local recurrence, regional recurrence, predictors, subtype

## Abstract

**Background:**

Although fairly uncommon, loco-regional recurrence in breast cancer (BC) has major consequences for the patient. Several predictors for locoregional have been previously reported from large randomized clinical trials mainly from Europe & North America; data from other geographical areas are somewhat scarce. Here we performed a retrospective review of medical records in a single academic center in Chile, searching for predictors of breast tumor recurrence.

**Results:**

Median patient follow up was 61 months, 5 year overall survival (OS) rate was 94.2% (95% CI 93–95.3). We found that 108 out of 2,754 (5.3%) patients had loco-regional recurrence. The 2-year loco-regional control was 98% (95% CI 97.3–98.7) and 5-year was 94% (95% CI 92.6–95.4). Univariate analysis showed a correlation between recurrence and being <50 year-old, positive surgical margins, advanced stage, subtype, and presence of LVI and omission of adjuvant radiotherapy. Only the absence of adjuvant RT was predictor of locoregional recurrence in multivariable (*p* **<** 0.001).

**Conclusions:**

Our study population presents high local control of BC. Age, surgical margins, stage, molecular subtype and absence of adjuvant radiotherapy were associated with loco-regional recurrence. Prospective trials and long-term follow up are required in order to confirm these results.

**Materials and Methods:**

We analyzed medical records from 2,201 BC patients at the Pontificia Universidad Católica de Chile from 1997 to 2016. Collected data included: age at diagnosis, tumor size, axillary involvement, molecular subtype, margin status, histological grade, lympho-vascular invasion (LVI) and ipsilateral recurrence.

## INTRODUCTION

For decades, mastectomy has been both the most common surgical treatment for stage I/II breast cancer (BC) and the preferred treatment for loco-regional disease. Alternatively, treatments may also consist in a combination of breast conserving surgery (BCS) plus radiotherapy (RT). Indeed, several prospective randomized clinical trials have demonstrated that this combination approach provides local control and patient survival rates equivalent to those observed with mastectomy [1].

Nevertheless, about 17% of patients treated with BCS plus RT develop ipsilateral breast tumor recurrence (IBTR) within 20 years of treatment [2]. The IBTR risk is the highest during the first 5 years following treatment, with an incidence rate of 5–10% [3]. Furthermore, patients that developed IBTR or loco-regional recurrence (LRR) have a significantly poorer prognosis [4].

Over the last decades, significant advances in systemic treatments along with a new classification of BC subtypes have increased the interest for predictors of recurrence in BC patients that have received treatments.

Several studies conducted in Europe and North America have analyzed clinical and histopathologic factors associated to an increased risk of BC recurrence. However, to the best of our knowledge such studies are yet to be reported in the Chilean population. Therefore, the aim of our study was to identify risk factors for loco-regional breast tumor recurrence in Chilean women with invasive BC.

## RESULTS

### Patient characteristics

**Figure 1:**
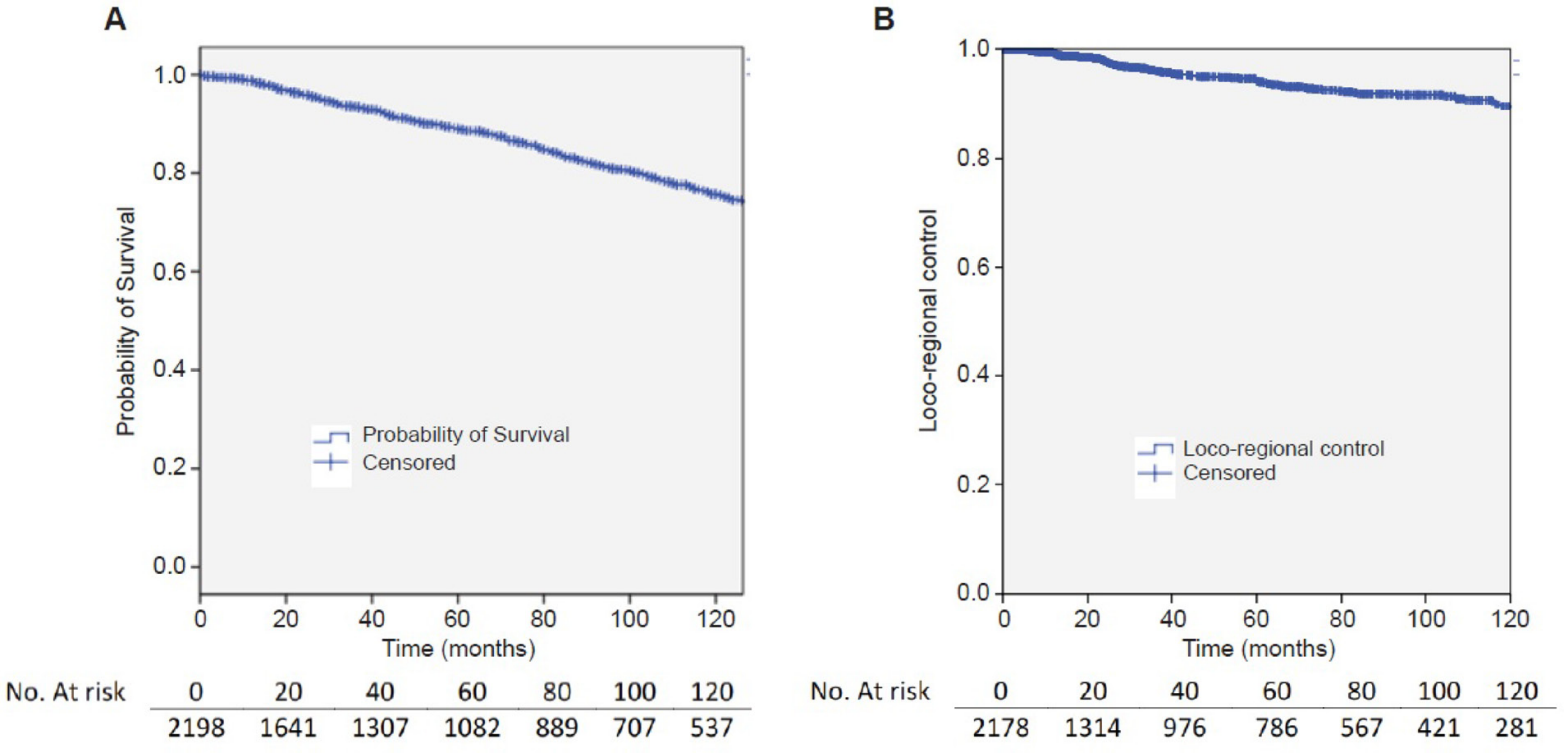
Overall survival rates (**A**) and loco-regional disease control (**B**).

A total of 2,754 patients were treated for BC in the period 1997–2016, within this group 2,201 had information about local control and were included into this study. Patient median follow up time for OS was 61 months. Median follow up for local control was 32.6 months (range 0–285). Main characteristics of patients, tumor and received treatments are summarized in Table 1. As shown in Figure 1A 5-year OS was 94.2% (95% CI 93–95.3).

**Table 1:** Patient, tumor and treatment characteristics

Characteristic	*N*	Median/(Range)
Age	2198	55/ (19-101)
**Characteristic**	***N***	**%**
**Breast surgery**		
Lumpectomy	1467	66.7
Mastectomy	593	26.9
Unknown	141	6.4
**Axillar surgery**		
SLND	985	44.8
AD	1016	46.2
Unknown	200	9.1
**Tumor Stage**		
1	814	37
2	804	36.5
3	367	16.7
4	46	2.1
Unknown	170	7.7
**Tumor Subtype**		
Luminal A	951	43.2
Luminal B	585	26.6
HER2-enriched	117	5.3
Triple-negative	225	10.2
Unknown	323	14.7
**Margins**		
Positive	90	4.1
Negative	671	30.5
Unknown	1440	65.4
**Vascular invasion**		
Positive	540	24.5
Negative	563	25.6
Unknown	1098	49.9
**Adjuvant Chemotherapy**		
No	857	38.9
Yes	897	40.8
Unknown	447	20.3
**Adjuvant Radiotherapy**		
No	185	8.4
Yes	1375	62.5
Unknown	641	29.1
**Adjuvant Hormonotherapy**		
No	285	12.9
Yes	1005	45.7
Unknown	911	41.4

Abbreviations: SLND: sentinel lymph node dissection. AD: axillar dissection.

### Locoregional control

Figure 1B shows that 2-year LRC was 98% (95% CI 97.3–98.7) and 5-year was 94% (95% CI 92.6–95.4). Overall, 108 out of 2,198 (5.3%) patients displayed BC recurrence, with a median time of recurrence: 66 months after surgery. The site of recurrence was available in 50 cases, and the most frequent site was the same quadrant of the breast (*N* = 18), followed by chest wall (14), and the axila (8), SCV was the first local recurrence in 6 patients, 3 presented a recurrence in the same breast but in a different quadrant and 1 had an internal mammary recurrence.

### Predicting loco-regional recurrence

**Figure 2:**
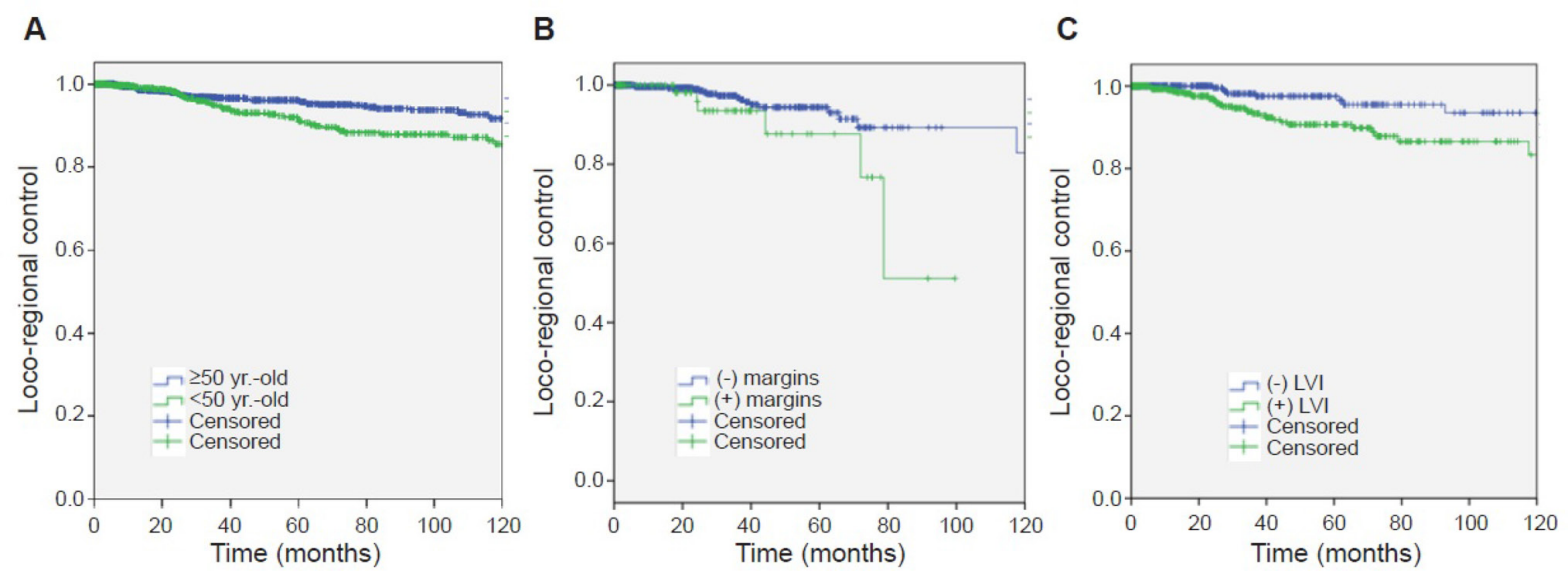
Loco-regional control of disease (**A**) by Age. (**B**) by Margin status, and (**C**) by lymphovascular Invasion (LVI).

A summary of the results of an univariate analysis for loco-regional recurrence is shown in Table 2. Briefly, loco-regional control at 2 and 5 years were 98% (95% CI 96.8–99.1) and 91% (95% CI 88–93.9) respectively for patients aged <50. For patients that were ≥50 years these values were 98% (95% CI 97–98.9) and 96% (95% CI 94.6–97.4), respectively (Figure 2A. *p* = 0.0013). Loco-regional control by surgical margin status is presented in Figure 2B. Univariate analysis showed that age, surgical margin status, lymphovascular invasion (LVI), breast cancer subtype, advanced stage (Stage III or IV) were predictors of regional recurrence.

**Table 2:** Univariate analysis

Variable	HR (IC)	*p*
Age <50	1.58 (1.096–2.28)	0.0013
Margin ±	2.57 (1.00–6.58)	0.049
Stage II vs I	1.62 (0.96– 2.76)	NS
Stage III vs I	2.98 (1.69–5.3)	<0.001
Stage IV vs I	12.98 (4.86–34.7)	<0.001
BCS vs MT	0.85 (0.57– 1.27)	NS
LV invasion +	2.99 (1.52– 5.9)	0.001
Luminal B vs LA	2.87 (1.64–5.05)	<0.001
Her2 + vs LA	5.88 (2.74–12.65)	<0.001
Triple neg vs LA	4.49 (2.3–8.5)	<0.001
Non-LA vs LA	3.56 (2.14–5.92)	<0.001
HT vs no HT	0.42 (0.27–0.65)	< 0.001
RT vs no RT	0.27 (0.17–0.42)	<0.001
No ACT vs ACT	1.0 (0.67–1.51)	NS

Abbreviations: BCS = breast conserving surgery, MT = Mastectomy, LV = lymphovascular invasion, LA = Luminal A, Triple neg = Triple negative, Non-LA = non luminal A subtype (LB, triple neg and Her2+ combined) HT = Hormonal therapy, RT = Radiotherapy, ACT = Adjuvant chemotherapy.

A Kaplan Meier analysis is shown in Figure 2C. LRR rates at 2 and 5 years were 99% (95% CI 98.2–99.7) and 97% (95% 94.8–99.1) without LVI; and 97% (95% CI 95.2–98.7) and 90% (95% CI 86.2–93.7) respectively with LVI (*p* = 0.001).

**Figure 3:**
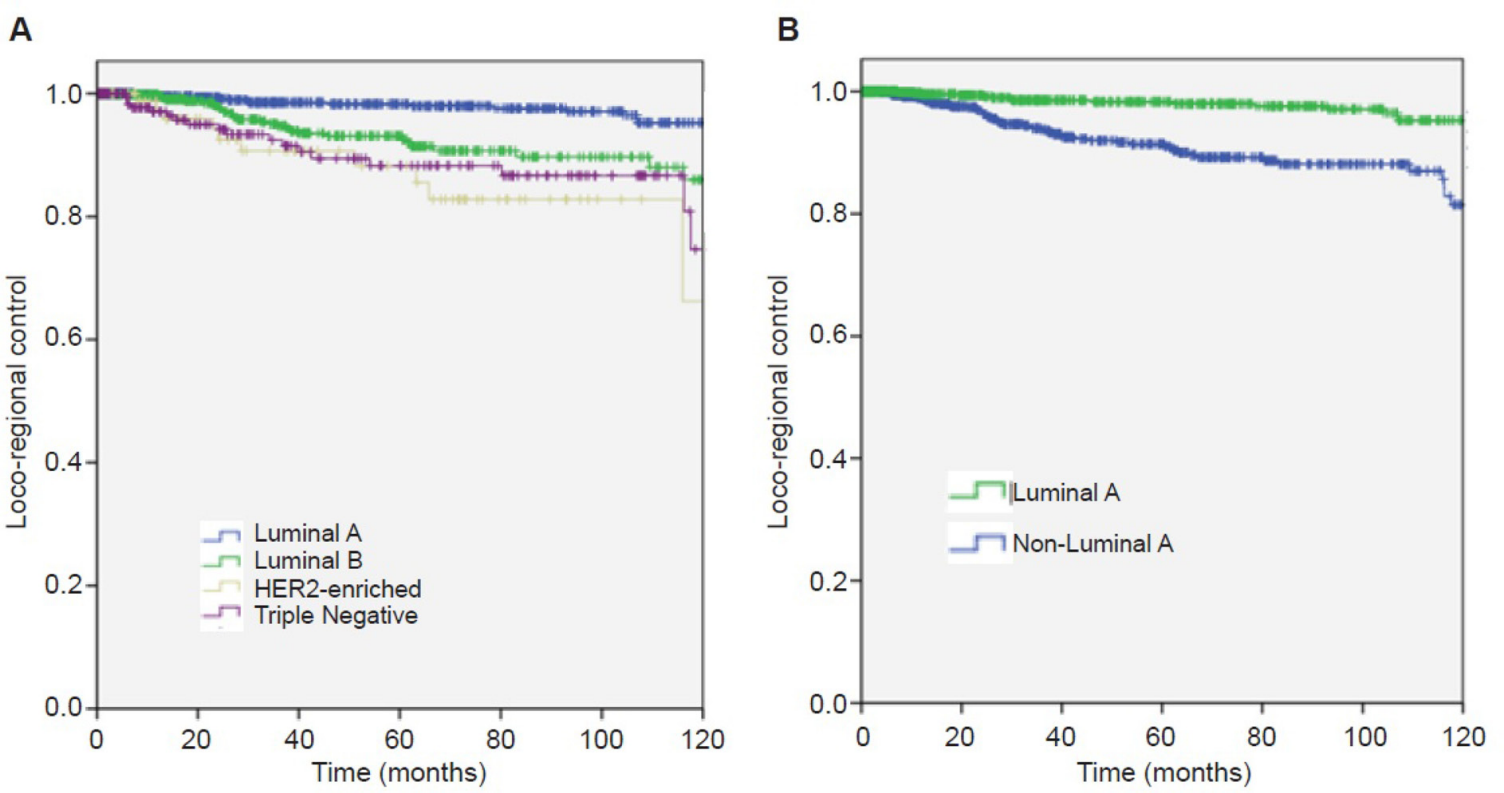
Loco-regional control of disease (**A**) by breast cancer subtype. (**B**) by breast cancer subtype comparing luminal A versus non-luminal A.

Next, LRC rates according to BC subtypes were analyzed. Within the luminal A subtype LRC rates at 2 and 5 years were 99.4% (95% CI 98.8–99.9) and 98.3% (95% CI 97.1–99.4) respectively. For luminal B, LRC at 2 and 5 years were 94.6% (95% CI 96–99.1) and 93.1% (95% CI 90.1–96). For HER2 enriched, LRC at 2 and 5 years were 92.5% (95% CI56–98.9) and 88.2% (95% CI 79.7–96.6). Finally, for triple negative, LRC rates at 2 and 5 years were 94.9% (95% CI 91.3–98.4) and 88.3% (95% CI 82.6–93.9), (Figure 3A).

Compared with Luminal A type, we obtained significant difference for all the subtypes (*p* < 0.001). With Luminal B (HR 2.875; 95% IC 1.64–5.05), HER2 positive (HR 5.88; 95% CI 2.74–12.65), triple negative (HR 4.496; 95% CI 2.3–8.5) *p ≤* 0.001. If grouped together, Luminal A compared with non Luminal A (HR 0.281; 95% CI 0.17–0.47; *p* < 0.001) favors Luminal A type *p* < 0.001, Figure 3B.

**Figure 4:**
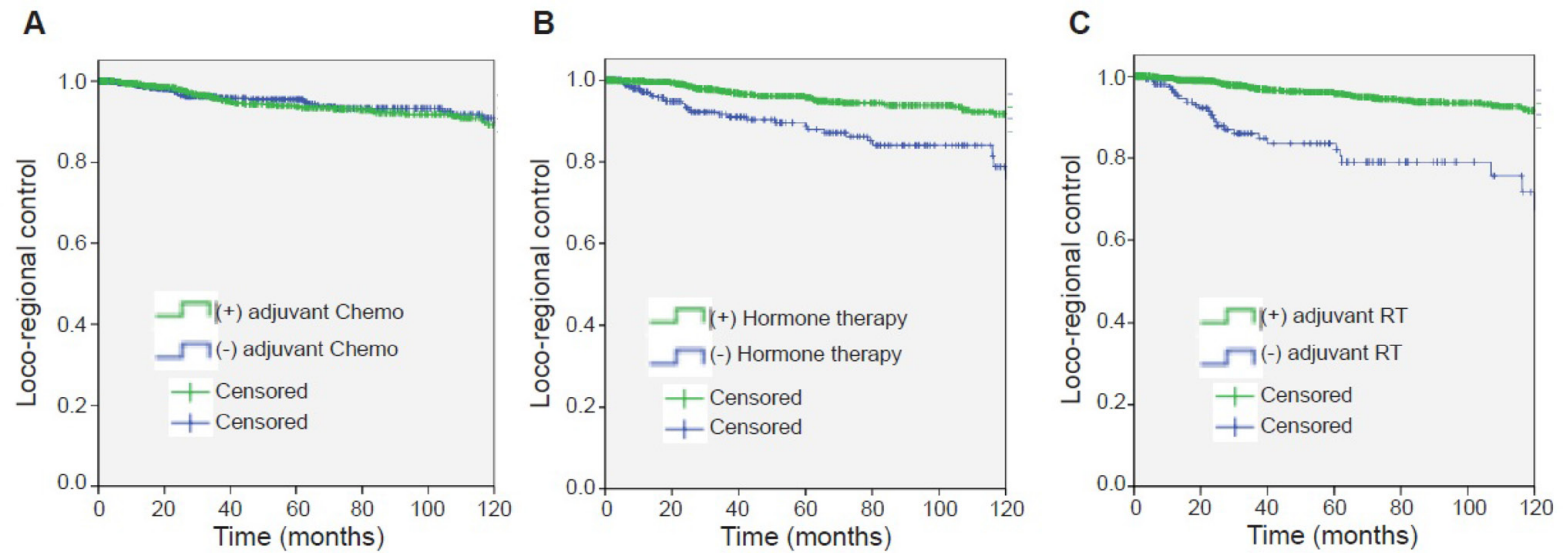
Loco-regional control of disease (**A**) in patients with or without adjuvant chemotherapy. (**B**) in patients with or without hormone therapy. (**C**) in patients with or without adjuvant radiotherapy (RT).

Notably, no association was found between risk of loco-regional recurrence and type of surgery (*p* = 0.43) or adjuvant chemotherapy (*p* = 0.978, Figure 4A). Conversely, adjuvant hormonotherapy (HR 0.418; 95% CI 0.27–0.65; *p* < 0.001, Figure 4B) or adjuvant RT 0.27 (0.17–0.42) *p* < 0.001, Figure 4C), were associated to better disease control. Patients at risk by subgroup is presented in Supplementary Table 1.

Patient who didn't receive adjuvant hormone therapy had higher LRC than those who did. The LRC rate at 2 and 5 years were 93.8% (95% CI 90.6–96.9%) and 88.8% (95% IC 84.2–93.3%) and 98.9% (95% CI 98.1–99.6) and 95.8% (95% CI 94.2–97.3) respectively *p* < 0.001. Adjuvant RT, also favored LRC control. The LRC at 2 and 5 years were 89.7% (95% CI 84.6–94.8) and 83.6% (95%CI 76.7–90.4) for those without RT and 98.8% (95% CI 98.2–99.4) and 95.8% (95% CI 94.4–97.1) for those with adjuvant RT *p* < 0.001.

The use of adjuvant chemotherapy was not associated to local recurrence (*p* = 0.978).

Finally, variables that were statistically significant (*p* < 0.05) were then entered into the multivariate analysis, however as shown in Table 3 only the absence of adjuvant RT maintained statistical significance: (HR 6.588: 95% CI 2.1–19.9; *p* < 0.001).

**Table 3:** Multivariate analysis

Multivariate	*p*	HR (95% CI)
Age < 50 years	0.823	1.1 (0.4–2.8)
Positive surgical margins	0.2	2.163 (0.6–7.1)
Positive lymphovascular permeations	0.213	2.171 (0.6–7.3)
Stage I vs.	—	—
Stage II	0.923	1.06 (0.3–4)
Stage III	0.099	3.246 (0.8–13.1)
Stage IV	0.152	5.45 (0.5–55–5)
Luminal A	0.193	0.482 (0.2–1.4)
No adjuvant RT	0.001	6.588 (2.1–19.9)

## DISCUSSION

In this study, we evaluated risk factors that determine loco-regional BC recurrence in a Chilean group of patients. To the best of our knowledge this is the first and the largest study of its kind in Chile.. Our study covers a long period of time of almost two decades (19 years), over this period we have seen significant advances in BC treatment. Evidently, local recurrence is a rather infrequent event, unless it is measured over a longer time period in order to capture a larger number of events, thereby introducing some heterogeneity into the collected data. Indeed, first we explored potential differences in loco-regional recurrence over two time periods 1997–2004 vs 2004–2016 and we did not find significant differences (not shown, Log rank 0.197).

Our data show that 5-year LRR control rate was 94%, similar to previous reports [5]. Also in agreement with previous studies loco-regional control was better in older patients (>50 year-old) [6–7]. Similarly, positive surgical margin status is commonly associated to an increase in the risk of, ipsilateral breast recurrence (HR 2.51) [8], and poorer prognoses.

Regarding BC subtypes, HER2+ was associated to higher LRR rates, again confirming published results from the literature [9–10] and suggesting a poor response of this subtype to conventional therapies. However, the use of trastuzumab in these patients improves clinical outcomes and increases the sensitivity to RT [11–12]. Our institution incorporated trastuzumab in 2011 when it became covered by medical insurance in our country.

Previous studies have suggested a relationship between BC subtypes and response rate to RT. Indeed, Luminal A is usually associated to better response; in contrast HER2+ cancers are considered less responsive to RT [13]. Despite this, a recent randomized trial demonstrates that BC subtype was not predictive of RT response, similar to our results (not shown).

Along the years of the study, RT was administered using two regimens: standard treatment, that consists of a 50 Gy dose in 25 daily fractions over 5 weeks; or hypofractionated, consisting on 15–16 fractions over 3 weeks, with or without a boost. Adjuvant RT in our study had an impact on loco-regional control, HR 0.269 (95%CI 0.172–0.42), as reported previously [14]. The most frequent sites of loco-regional recurrence were the breast and the chest wall followed by the supraclavicular and the axilla. One patient displayed regional recurrence in internal mammary.

Our univariate analysis showed significant associations between age, surgical margin, stage, molecular subtype, LVI status and adjuvant RT with LRR but multivariate analysis was the only variable significantly associated with local recurrence. Despite these results, our findings must be interpreted cautiously, mainly due to a relatively small number of cases/events and the amount of missing/lost data. In addition to this, our study has a number of other limitations: first, it is a retrospective study based at a single center, second the recruiting period time was long enough (almost 20 years) to experience changes in classifications and standard treatments, the incorporation of subtype was based on modified UC criteria, third biopsies were done by local pathologists and we did not have a centralized pathology review. Some patient information regarding chemotherapy schemes, hormone therapy and RT was either not available or highly heterogeneous in some cases. Finally, there is a possibility that some patients might have had follow ups or receive treatments in other medical centers, in these cases recurrent disease could not have been recorded, all these limitations have to be considered and therefore the interpretation of our results should be done with caution.

## MATERIALS AND METHODS

### Study design

This was a retrospective study of the medical records of invasive BC patients treated between 1997 and 2016 at the Pontificia Universidad Católica de Chile and the Red de Salud UC Christus.

Our study was performed in compliance with the Declaration of Helsinki. All procedures, along with permission to access the databases were approved by the local research ethics committee at the Pontificia Universidad Católica de Chile. Due to the retrospective and therefore non-interventional nature of this study no written informed consent were requested by the local research committee.

Patients were included if they were treated for invasive BC regardless of histology type, or any kind of surgery (Mastectomy or BCS) for their primary and adjuvant treatments according to local guidelines. Patients with no information on loco-regional BC recurrence were excluded.

Patient characteristics were assessed at the time of diagnosis and included: age, tumor size, axillary involvement, margin status (negative margin was defined by no ink on tumor) and lympho-vascular invasion.

Pathological reports from the primary tumor were reviewed regarding histological type, tumor size, nodal compromise and histological grade, according to Elston & Ellis [15]. Receptor status was determined via immunohistochemistry. Estrogen and progesterone receptors (ER and PR respectively) were defined as positive when ≥1% of tumor cells showed nuclear positive staining. Tumors that had a HER2 score of 3+ were considered HER2+ [16]. For HER2 2+, a fluorescence *in situ* hybridization study was done. Since in our center Ki67 is not routinely indicated, it was excluded from our analyses. Tumor stage was determined according to the guidelines of the American Joint Committee on Cancer 7th [17]. Tumors were classified into 4 subtypes according to immunohistochemistry markers and histological grade, as described [18]. Luminal A (ER+ and/or PR+, HG 1–2, HER2-), Luminal B (ER+ and/or PR+, HG 3 and/or HER2+), triple negative (ER-, PR- and HER2-) and HER2-enriched (ER- and PR-, HER2+).

Patients were treated according to local guidelines. Briefly, early stage BC were treated with BCS and RT. Loco-regional advanced BC were treated with mastectomy with or without adjuvant RT or neoadjuvant chemotherapy followed by breast surgery and RT. Patients with clinically negative axilla has sentinel lymph node dissection, if this was positive, patient has axillary dissection. Radiotherapy dosing was 50 Gy in 25 fractions to the breast/chest wall ± regional lymph nodes if indicated. Boost was 10 Gy in 5 fractions. Since 2010 an increasing percentage of early stage BC have been treated with hypofractionated RT of 42.5 Gy in 16 fractions.

All patient cases were discussed at a breast multidisciplinary board in order to decide the best adjuvant treatment. Chemotherapy was recommended for patients with a >1.0 cm diameter triple negative BC, and for ER+ BC with extensive positive axilla. Trastuzumab was recommended for HER2+ BC. Radiotherapy was indicated for patients with BCS and patients with mastectomy and + lymph nodes.

Follow-up time started at the day of diagnosis. Patients were followed-up every three months during the first year, every four months during the second year, and every six months at the third to fifth year and then yearly. Clinical examination was performed in every visit. Mammography was performed yearly for patients with BCS, breast ultrasound was added for patients with a dense breast.

### Statistical analysis

Loco-regional failure was defined as any evidence of recurrence in the same breast or regional lymph nodes (including axillary, supraclavicular or internal mammary). The Kaplan–Meier method was used for survival analysis. For univariate analysis log rank test was used. Exploratory subgroup analysis was done for age (<50 years), positive margins, vascular invasion, AJCC stage, molecular surrogate subtype, adjuvant hormonal therapy, adjuvant chemotherapy, type of breast surgery

We used Cox proportional hazard regression model for multivariate analysis. Data analysis was performed using IBM SPSS version 19.

## CONCLUSIONS

To date, this is the largest study that assesses BC recurrence risk factors and OS in Chilean women. Our data obtained from an academic center suggest a high loco-regional control rate in patients (>94 %). As expected, patients that were: <50-year-old, had positive surgical margins, advanced stage, a non-Luminal A subtype, had LVI or did not receive adjuvant RT had an increased risk for LRR. Future prospective and multicentric studies with long-term follow up periods should expand and confirm our findings.

## SUPPLEMENTARY MATERIALS FIGURES AND TABLES


