# Supplementary material for: Risk factors for loco-regional recurrence in breast cancer patients: a retrospective study
Source: Oncotarget. 2018 Jul 13;9(54):30355–62. doi: 10.18632/oncotarget.25735 (PMC6084401; doi:10.18632/oncotarget.25735)
Supplement: Supplementary file 1 [file oncotarget-09-30355-s001.pdf]

Risk factors for loco-regional recurrence in breast cancer patients: a retrospective study

SUPPLEMENTARY MATERIALS

Supplementary Table 1: Patients at risk by subgroup

Patients at risk	Months					
	**0**	**12**	**24**	**36**	**48**	**60**
Overall Survival	2198	1826	1561	1368	1232	1082
Loco-Regional control	2178	1476	1229	1037	891	785
Age						
LC < 50	749	523	434	363	307	266
LC ≥ 50	1426	955	793	672	583	515
Margins						
+ Margins	90	53	42	24	12	9
– Margins	668	372	272	182	119	77
Lymphovascular invasion						
–LVI	561	561	225	167	129	101
+LVI	424	367	273	206	150	120
BC subtype						
Luminal A	947	612	508	427	372	321
Luminal B	577	415	325	254	202	171
HER2-enriched	114	67	55	47	37	34
Triple Negative	223	146	120	99	83	72
Chemotherapy						
–Chemo	851	563	455	370	324	281
+Chemo	886	732	619	529	444	391
Hormonal therapy						
–Hormone therapy	283	218	181	153	126	111
+Hormone therapy	996	865	740	622	531	463
Radiotherapy						
–RT	184	134	103	72	65	57
+RT	1360	1177	1016	880	760	670
